# Association of non-high-density lipoprotein cholesterol to high-density lipoprotein cholesterol ratio and atherogenic index of plasma with obstructive sleep apnea

**DOI:** 10.3389/fpsyt.2025.1597820

**Published:** 2026-01-12

**Authors:** Guilian Li, Mingzhu Deng, Guohua He, Yan Jiang, Zhen Wang, Cai Zhou, Wei Xu, Tieqiao Feng, Wengao Zeng, Jian Peng, Ling Xiao, Dandan Yang, Kangping Song, Fangyi Li

**Affiliations:** 1 Department of Neurology, The Affiliated Changsha Central Hospital, Hengyang Medical School, University of South China, Changsha, Hunan, China; 2 Department of Neurology, The Second People’s Hospital of Hunan Province (Brain Hospital of Hunan Province), Changsha, Hunan, China; 3 Department of Rehabilitation, The Affiliated Changsha Central Hospital, Hengyang Medical School, University of South China, Changsha, Hunan, China

**Keywords:** apnea-hypopnea index, atherogenic index of plasma, lipid abnormality, non-high-density lipoprotein cholesterol to high-density lipoprotein cholesterol ratio, obstructive sleep apnea

## Abstract

**Background:**

Obstructive sleep apnea (OSA) is a prevalent sleep disorder associated with metabolic, cardiovascular disorders. The non-high-density lipoprotein cholesterol to high-density lipoprotein cholesterol ratio (NHHR) and atherogenic index of plasma (AIP) are thought to be better ways to detect cardiovascular diseases than single lipid parameters. The relationship between NHHR or AIP and OSA remains ambiguous.

**Methods:**

454 consecutive patients with suspected OSA were enrolled between June 2023 and February 2025. The OSA was defined as the occurrence of more than 5 apnea-hypopnea index (AHI) events/h. Spearman’s rank correlation analysis among the NHHR, AIP, AHI, and lowest SpO_2_. Binary logistic regression analysis was conducted to investigate the association between the NHHR and AIP with OSA. To evaluate the diagnostic performance of the NHHR and AIP for OSA, we conducted receiver operating characteristic (ROC) curve analysis.

**Results:**

Among the 454 recruited patients, 318 (70.04%) were diagnosed with OSA. A binary logistic regression model showed that the NHHR (odds ratio [OR], 1.559; 95% CI 1.268–1.915, *p<*0.001) and AIP (OR, 1.349; 95% CI 1.146–1.843, *p* < 0.001) were independent factors for OSA. The area under the curve (AUC) values for the identifying OSA were 0.730 for the NHHR, 0.658 for the AIP and 0.763 for the combined model, respectively.

**Conclusions:**

Our study indicates the NHHR and AIP were independent risk factors for OSA and can be used as potential tools for OSA.

## Introduction

Obstructive sleep apnea (OSA) is defined by recurrent episodes of complete or partial upper airway obstruction during sleep, resulting in intermittent hypoxemia and sleep fragmentation (1). Epidemiological studies estimate that obstructive sleep apnea (OSA) affects approximately 9% to 38% of the general population (2). Symptoms of OSA encompass excessive diurnal somnolence, neurocognitive deficits, reduced quality of life, along with endocrine, metabolic, and cardiovascular alterations (1, 3, 4). If OSA fails to be managed, it can lead to major health issues like high blood pressure, metabolic syndrome, diabetes, and cardiovascular illnesses (5–7). Untreated OSA has been associated with approximately $150 billion in additional health care and other costs per year (8). Polysomnogram is the gold standard for diagnosis of OSA. However, several hospitals lack sleep facilities and are incapable of doing polysomnography examinations. Consequently, the identification of modifiable risk factors and quantifiable biomarkers in patients with OSA is therefore crucial for enhancing early detection, risk stratification, and targeted intervention strategies.

A substantial body of evidence has elucidated the intricate interplay between dyslipidemia and OSA, revealing complex, bidirectional pathophysiological interactions (9–12). Recently, compared to isolated lipid measures, the non-high-density lipoprotein cholesterol to high-density lipoprotein cholesterol ratio (NHHR) and the atherogenic index of plasma (AIP) are thought to be more accurate indicators of cardiovascular illnesses (10, 11, 13). The NHHR has been identified as a comprehensive marker for assessing atherosclerosis since it integrates characteristics of both HDL-C and non-HDL-C. In comparison to conventional lipid indicators, prior studies have shown its higher predictive and diagnostic performance in determining the risk of atherosclerosis (14), type 2 diabetes (15), and metabolic syndrome (16). Furthermore, prior cross-sectional studies indicate that NHHR could serve as a potential instrument for predicting OSA (10, 13). Nonetheless, it remains uncertain whether the same conclusion is applicable to the Asian population. The AIP is determined by the logarithmic ratio of triglyceride (TG) to high-density lipoprotein cholesterol (HDL-C) levels (17). AIP has been utilized to assess metabolic syndrome, insulin resistance, and atherogenic dyslipidemia (16–18). Previous studies have shown an increase in AIP levels in OSA and a correlation with the severity of the disease. Nonetheless, its clinical significance remains limited (11).

Currently, numerous risk variables, including male gender, elevated baseline body mass index, asthma, a particular genetic variant at rs12415421, and insulin resistance/hyperglycemia, have been discovered by researchers as connected with OSA (19). Nevertheless, the relationship between NHHR and AIP with OSA requires additional examination.

## Methods study design and subjects

**Figure 1:**
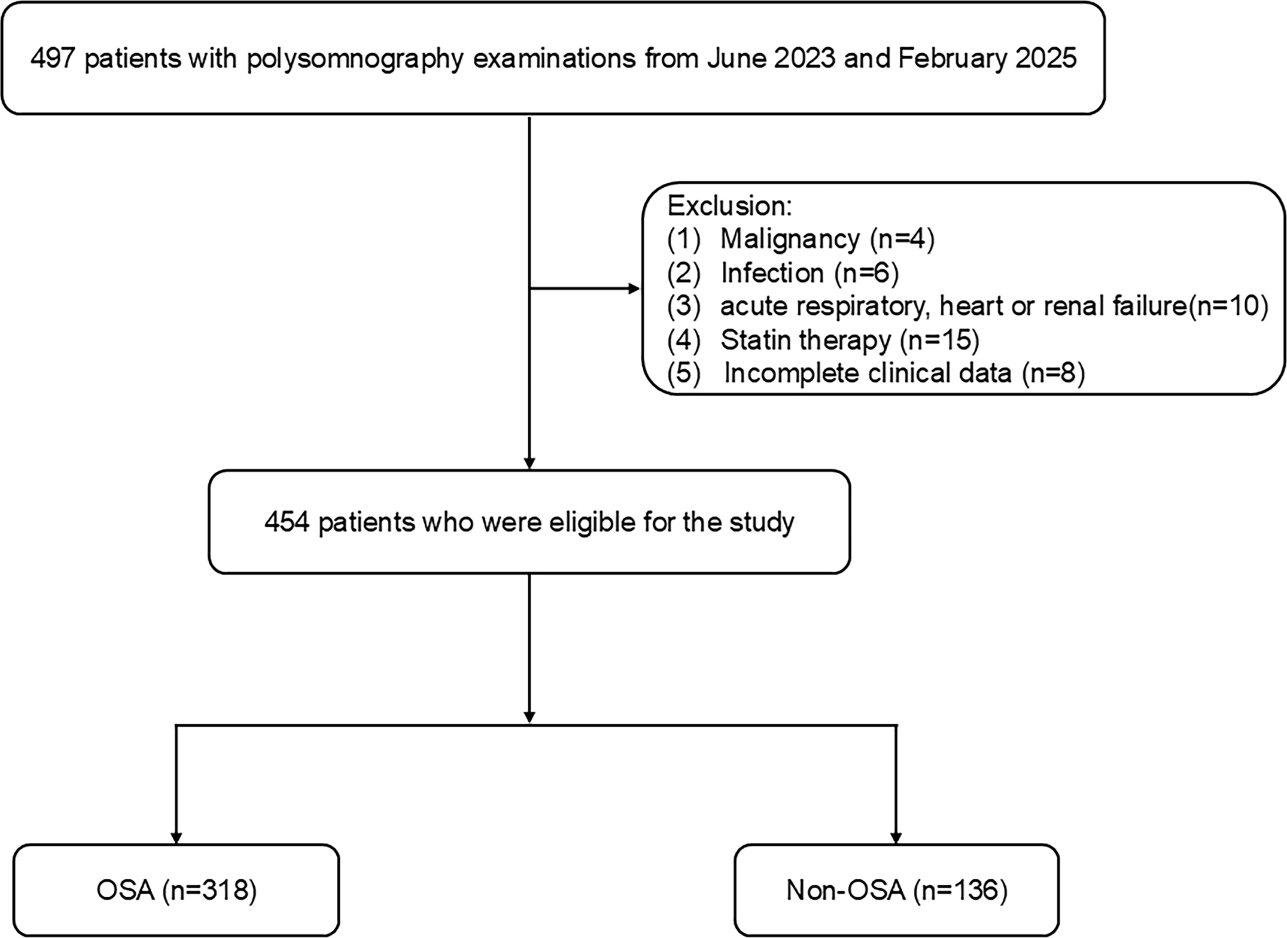
Study flow diagram. OSA, obstructive sleep apnea.

Study participants were retrospectively enrolled from Changsha Central Hospital between June 2023 and February 2025, comprising consecutive patients meeting the predefined eligibility criteria. The study protocol received formal approval from the Ethics Committee of Changsha Central Hospital, and the official approval number is CS0014. Inclusion criteria were defined as follows (1): The patient exhibited at least one indicator of OSA, including nocturnal choking, snoring, heightened daytime somnolence, and witnessed apneic episodes (2); none of them had ever been diagnosed with OSA before, nor had they ever undergone any kind of treatment for the condition, including upper airway surgery, mandibular advancement devices, or continuous positive airway pressure; and (3) participants who were at least 18 years old and had a minimum of four hours of sleep. Exclusion criteria included (1): The patients with malignancy within 10 years (2); infection within 2 months (3); autoimmune disorders (4); acute heart disease and respiratory failure (5); nasal polyp; and (6) on lipid-modifying therapy. Every research participant had an overnight polysomnographic assessment with the iRem-A system (Physio Med, Hangzhou, CHINA). Patient data was collected utilizing surface electrodes for electrooculography, electrocardiography, electroencephalography, and electromyography. Furthermore, oral and nasal ventilation, abdominal and thoracic movement, and tracheal sounds were simultaneously recorded. Transcutaneous peripheral oxygen saturation (SpO_2_) was continuously monitored using a pulse oximeter. Alterations in body position during sleep were also documented in the study. After the data was collected using a computerized polysomnographic device, a manual scoring procedure was implemented. In our investigation, 454 patients were recruited. **Figure 1** illustrates an exhaustive flow diagram for patient enrollment.

### Clinical assessment and subject grouping

The slumber state was characterized using the criteria of Rechtschaffen and Kales. The criteria established by the American Academy of Sleep Medicine were used to evaluate the respiratory events. Apnea was defined as a decrease in ventilation of ≥ 90% of baseline values for a duration of ≥ 10 seconds. Hypopnea was defined as an event characterized by a ≥ 30% reduction in ventilation for ≥ 10 s and a ≥ 3% decrease in oxygen saturation or alertness. The apnea-hypopnea index (AHI) was defined as the number of apnea and hypopnea events that occurred per hour during sleep. OSA was defined in accordance with the International Classification of Sleep Disorders (Third Edition) criteria, which are as follows: an AHI of 5 or higher with daytime or nocturnal symptoms or the presence of comorbidities, or an AHI of 15 or higher regardless of symptoms or comorbidities. In order to categorize patients with OSA into three severity categories, the number of events per sleep night was employed: mild (AHI 5-15), moderate (AHI 16-30), and severe (AHI >30).

### Data collection

Demographic characteristics, clinical parameters, and polysomnographic data—including age, sex, body mass index (BMI), comorbidities (diabetes mellitus, coronary artery disease, hypertension, atrial fibrillation), health behaviors (smoking, alcohol consumption), and sleep study metrics (lowest SpO_2_, sleep latency, sleep efficiency)—were systematically documented for all participants. BMI was calculated as weight in kilograms divided by height in meters squared (kg/m²). Diabetes mellitus was identified by fasting glucose ≥126 mg/dL, use of glucose-lowering agents, or prior medical diagnosis. Hypertension was defined as systolic/diastolic blood pressure ≥140/90 mmHg, current use of antihypertensive medication, or previously established diagnosis. Atrial fibrillation was confirmed by documented electrocardiographic evidence of characteristic irregular rhythm or clinical history thereof. Coronary artery disease was defined as a history of myocardial infarction, revascularization, angiographically confirmed stenosis, or previous diagnosis. Smoking status was categorized as current smoking if the participant consumed ≥10 cigarettes daily for at least five years prior to enrollment. Similarly, alcohol consumption was defined as regular intake of ≥20 grams of ethanol per day maintained for a minimum of five years.

Fasting venous blood samples were obtained from all participants between 6:00 and 7:00 a.m. following a minimum 8-hour overnight fast. Complete blood count analysis was performed using an automated hematology analyzer (BZ6800, China) to quantify white blood cells (WBC), hemoglobin (Hb), and neutrophil counts. Biochemical profiling was conducted with an automated analyzer (HITACHI 7600, Japan) to measure low-density lipoprotein cholesterol (LDL-C), high-density lipoprotein cholesterol (HDL-C), total cholesterol (TC), triglycerides (TG), and fasting blood glucose (FBG). Two derived indices were calculated from these measurements: NHHR was determined as (TC - HDL-C)/HDL-C, AIP was computed using the formula Log [TG (mmol/L)/HDL-C (mmol/L)].

### Statistical analysis

Statistical analyses were performed using SPSS 25.0 (IBM Corp., Armonk, NY, USA) and MedCalc 15.6.0 (MedCalc Software, Ostend, Belgium). Data distribution was assessed using the Kolmogorov-Smirnov test. Continuous variables with normal distribution are presented as mean ± standard deviation (SD), while non-normally distributed variables are expressed as median (interquartile range). Categorical variables are summarized as frequencies and percentages. Group comparisons were performed using Chi-square or Fisher’s exact tests for categorical variables, and Student’s t-test or Mann-Whitney U test for continuous variables, as appropriate. Multicollinearity among independent variables was evaluated through collinearity diagnostics. The distributions of NHHR and AIP across OSA severity groups were visualized using box plots. Spearman’s rank correlation analysis was employed to examine the relationships between NHHR, AIP, and polysomnographic parameters (AHI and lowest SpO_2_). Binary logistic regression was used to identify independent risk factors for OSA. All statistical analyses were two-tailed, with a *p* < 0.05 considered statistically significant.

## Results

### Comparison of clinical and demographic characteristics between patients with OSA and non-OSA

**Figure 2:**
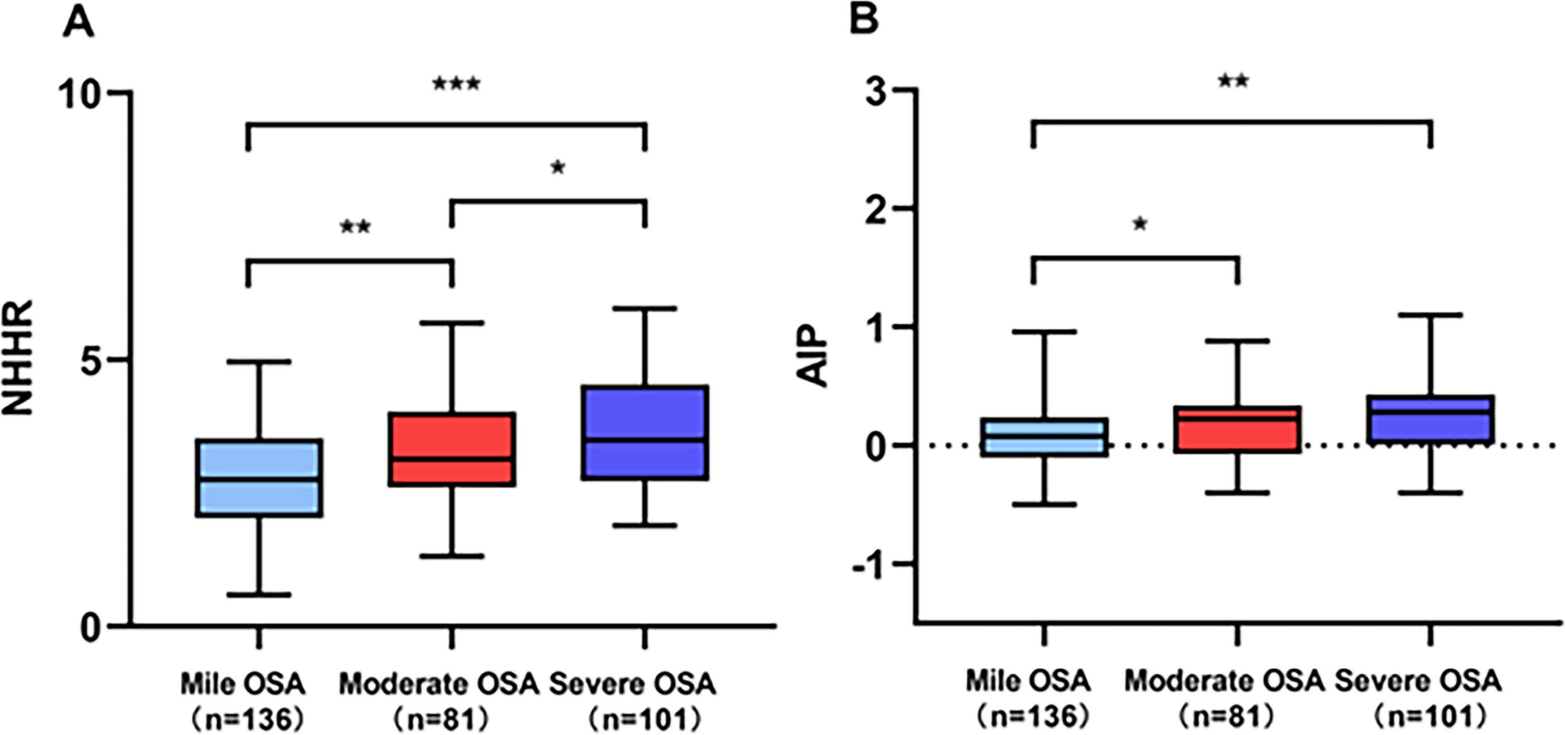
Comparison of **(A)** NHHR and **(B)** AIP across different severity levels of OSA. ***p < 0.001, **p < 0.01, *p < 0.05.

Baseline characteristics of the study participants are summarized in **Table 1**. Among the 454 enrolled patients, 318 (70.04%) were diagnosed with OSA, while 136 (29.96%) comprised the non-OSA group. Comparative analyses revealed significant differences between the two groups. The OSA group demonstrated significantly higher proportions of males (*p* < 0.001), BMI (*p* < 0.001), hypertension (*p* = 0.007), AHI (*p* < 0.001), NHHR (*p* < 0.001), and AIP (*p* < 0.001). Conversely, the OSA group showed significantly reduced levels of lowest SpO_2_ (*p* < 0.001), sleep latency (*p* = 0.026), and HDL-C (*p* = 0.02) compared to the non-OSA group. Furthermore, **Figure 2** portrays the comparison of the NHHR and AIP among OSA patients of varying severity.

**Table 1:** Baseline characteristics of the study population.

Variable	Total (n=454)	Non-OSA (n=136)	OSA (n=318)	*p*
Age, years	55.41 ± 15.34	51.82 ± 17.92	56.94 ± 13.83	0.002
Male, n (%)	349(76.87)	88(64.71)	261(82.08)	<0.001
BMI, kg/m^2^	25.48 ± 4.73	23.24 ± 3.79	26.44 ± 4.78	<0.001
Current smoking	186(40.97)	53(38.97)	133(41.82)	0.571
Current drinking	92(20.26)	25(18.38)	67(21.07)	0.514
Hypertension	286(63.00)	73(53.68)	213(66.98)	0.007
Diabetes mellitus	116(25.55)	29(21.32)	87(27.36)	0.177
Atrial fibrillation	72(15.86)	20(14.71)	52(16.35)	0.660
Coronary artery disease	93(20.48)	24(17.65)	69(21.70)	0.327
Lowest SpO2 (%)	84 (79–88)	88.50 (86–91)	81 (76–86)	<0.001
AHI (/h)	11.44(3.39-26.02)	1.34(0.43-2.79)	18.06(10.46-34.46)	<0.001
Sleep latency (min)	75(40.12-115.32)	80.52(54.83-150.12)	65.15(42-100.35)	0.026
Sleep efficiency (%)	69.32(54.79-81.64)	71.15(54.93-83.49)	66.82(54.77-80.44)	0.268
WBC (×10^9^/L)	6.93(5.88-8.19)	6.88(5.83-8.23)	6.94(5.90-8.19)	0.841
Hb(g/L)	136.03 ± 18.23	136.57 ± 16.86	135.80 ± 18.81	0.682
Neutrophils (×10^9^/L)	4.42(3.50-5.47)	4.40(3.51-5.50)	4.52(3.47-5.44)	0.766
FBG (mmol/L)	5.84(4.86-7.32)	5.76(4.85-7.27)	6.00(4.88-7.58)	0.499
TG (mmol/L)	1.34(0.94-1.95)	1.30(0.87-1.91)	1.36(0.96-1.97)	0.263
TC (mmol/L)	4.38 ± 1.01	4.36 ± 0.91	4.38 ± 1.05	0.868
HDL-C (mmol/L)	1.12(0.92-1.24)	1.18(0.96-1.24)	1.05(0.90-1.25)	0.020
LDL-C (mmol/L)	2.81(2.22-3.42)	2.80(2.22-3.47)	2.86(2.24-3.40)	0.890
NHHR	2.78(2.13-3.48)	2.49(1.94-3.04)	2.89(2.04-3.12)	<0.001
AIP	0.11(-0.29-0.27)	0.02(-0.13-0.17)	0.15(0.01-0.30)	<0.001

AHI, Apnea-hypopnea index; BMI, body mass index; WBC, white blood cell, Hb, hemoglobin; FBG, fasting blood glucose; TG, triglycerides; TC, total cholesterol; AIP, atherogenic index of plasma; NHHR, non-high-density lipoprotein cholesterol to high-density lipoprotein cholesterol ratio; LDL-C, low-density lipoprotein cholesterol; HDL-C, high-density lipoprotein cholesterol.

### The patients of OSA correlation analysis of the NHHR, AIP, AHI, and lowest SpO_2_

**Figure 3:**
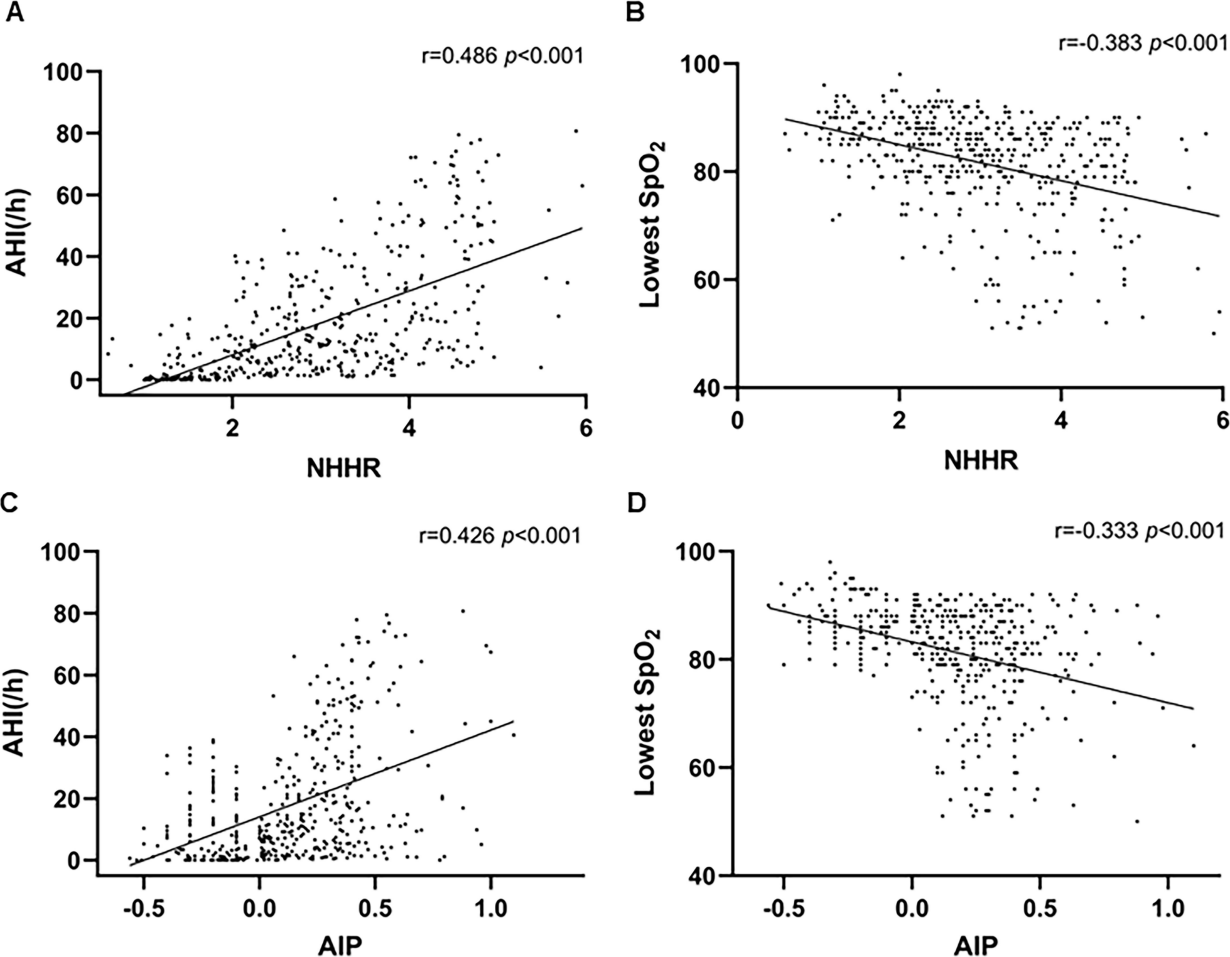
Positive correlations were observed between **(A)** NHHR and AHI (r = 0.486, *p* < 0.001) and **(C)** AIP and AHI (r = 0.426, *p* < 0.001). Negative correlations were observed between **(B)** NHHR and lowest SpO_2_ (r = -0.383, *p* < 0.001) and **(D)** AIP and lowest SpO_2_ (r = -0.333, *p* < 0.001).

We conducted Spearman’s rank correlation analysis among the NHHR, AIP, AHI, and lowest SpO_2_. We found that the NHHR (r=0.486, *p* < 0.001) and AIP (r=0.426, *p* < 0.001) were positively correlated with AHI. However, the NHHR (r=-0.383, *p* < 0.001) and AIP (r=-0.333, *p* < 0.001) were negatively correlated with the lowest SpO_2_ (**Figure 3**).

### Logistic regression analysis of risk factors for OSA

**Figure 4:**
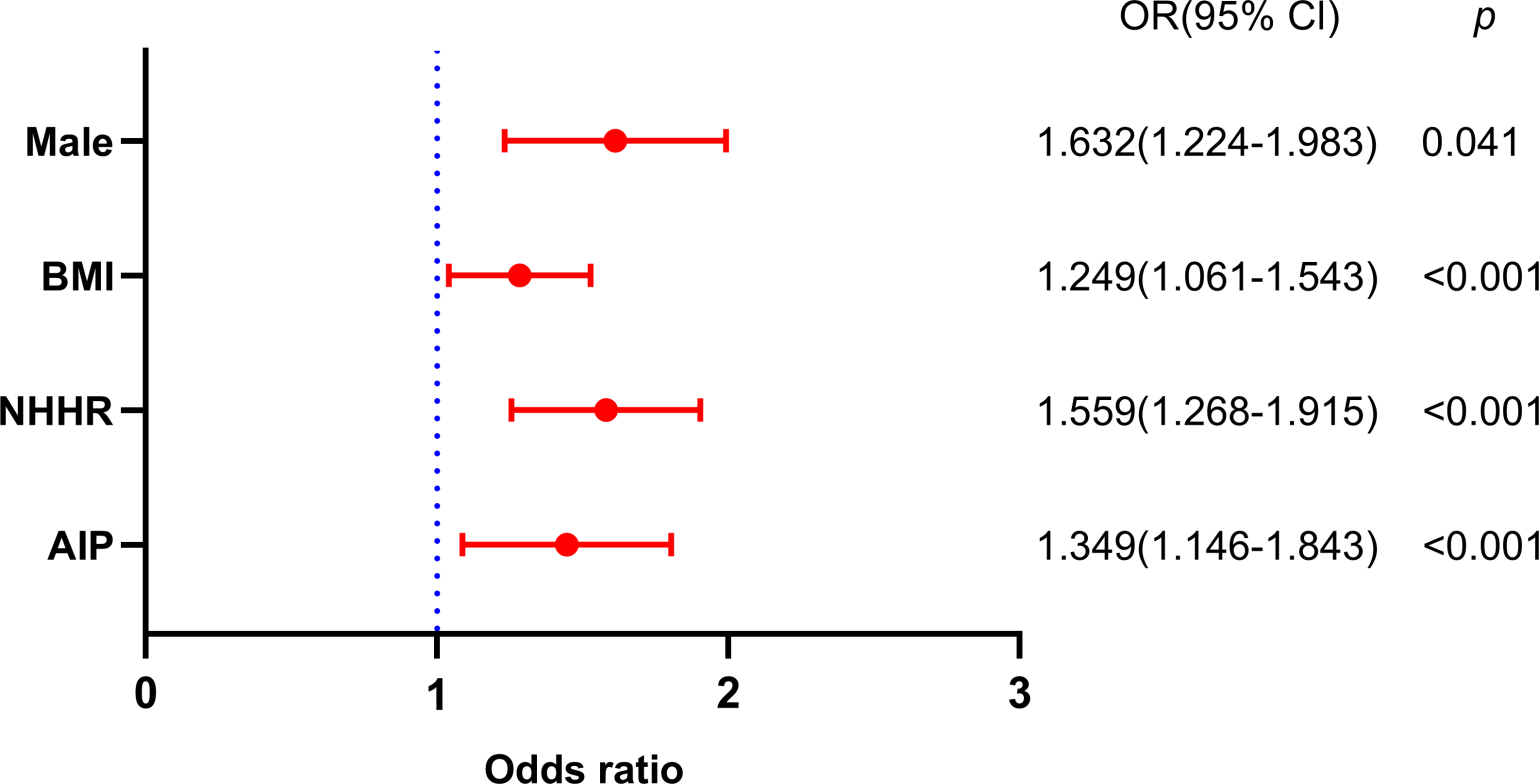
Binary logistic regression analysis of factors independently associated with OSA.

The OSA rudimentary models’ results are illustrated in **Table 2**. Variables demonstrating statistical significance in the univariate analysis (**Table 1**) were included in a binary logistic regression model to identify independent risk factors for OSA. Age is also a significant factor in OSA and should be incorporated into multivariate analysis. The use of variance inflation factor (VIF) values would be helpful for collinearity checking. Collinearity diagnostics indicated no significant multicollinearity between the AIP and NHHR, with VIF values of 2.12 and 1.56, respectively. Nevertheless, the HDL-C were excluded from the model as a result of their collinearity with the NHHR (VIF = 65) and AIP (VIF = 59). The male (OR, 1.632; 95% CI 1.224-1.983, *p* = 0.041), BMI (OR, 1.249; 95% CI 1.061-1.543, *p* < 0.001), NHHR (OR, 1.559; 95% CI 1.268–1.915, *p<*0.001), and AIP (OR, 1.349; 95% CI 1.146–1.843, *p* < 0.001) were identified as independent factors for OSA (**Figure 4**).

**Table 2:** Logistic regression analysis of risk factors for OSA.

Variable	OR (95% CI)	*p*	Adjusted OR (95% CI)	*p*
age	1.425(1.223-1.683)	0.098	1.223(1.152-1.515)	0.159
Male	2.489(1.587-3.931)	<0.001	1.632(1.224-1.983)	0.041
BMI	1.364(1.181-1.552)	<0.001	1.249(1.061-1.543)	<0.001
Sleep latency	0.945(0.764-0.986)	0.045	0.972(0.843-0.992)	0.402
Hypertension	1.102(1.015-1.346)	0.030	1.095(1.043-1.256)	0.290
HDL-C	0.453(0.212-0.843)	0.004		
NHHR	2.019(1.782-2.557)	<0.001	1.559(1.268-1.915)	<0.001
AIP	1.921(1.453-2.754)	<0.001	1.349(1.146-1.843)	<0.001

### Subgroup analyses and interaction test

Stratified analyses were performed to evaluate the consistency of the associations between NHHR, AIP, and OSA across predefined demographic and clinical subgroups (**Supplementary Figure 1**). The positive associations remained robust in all subgroups stratified by age (<65,≥65), gender (male vs. female), alcohol consumption, smoking status, coronary artery disease, atrial fibrillation, diabetes mellitus, and hypertension. Interaction tests confirmed no significant effect modification by any of these covariates (all *p* for interaction > 0.05). These findings demonstrate the robustness and broad generalizability of our conclusions across diverse population subgroups.

### ROC curve analysis is used to assess the overall discriminatory capacity of a diagnostic test for OSA

**Figure 5:**
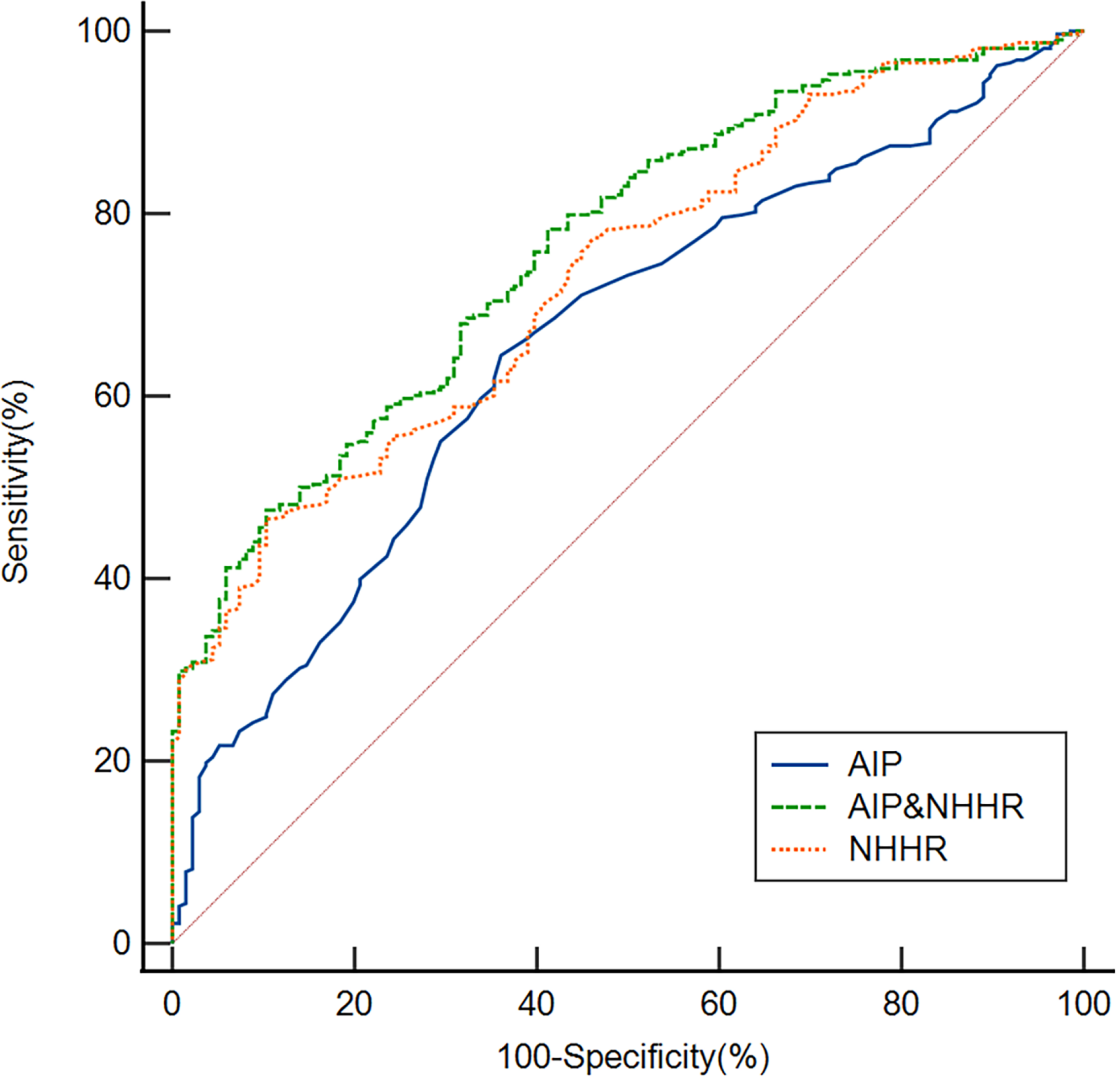
Based on ROC analysis, the AUC values for the NHHR, AIP, and their combination in identifying OSA were 0.730, 0.658, and 0.763, respectively.

ROC analysis was performed to evaluate the discriminative ability of the NHHR and AIP for OSA (**Figure 5**). The NHHR achieved an area under the curve (AUC) of 0.730 (95% CI: 0.687-0.771; *p* < 0.001), with an optimal cutoff value of 3.37 yielding a sensitivity of 46.54% and specificity of 89.71%. The AIP demonstrated an AUC of 0.658 (95% CI: 0.613-0.702; *p* < 0.001), with a cutoff of 0.07 corresponding to 64.47% sensitivity and 63.97% specificity. Notably, the combination of NHHR and AIP showed improved diagnostic performance (AUC = 0.763; 95% CI: 0.721-0.801; *p* < 0.001), with a cutoff of 0.79 providing 47.48% sensitivity and 89.71% specificity.

## Discussion

We gained several new insights from this study. Firstly, we found that the NHHR and AIP in OSA patients were higher than those in non-OSA patients. In addition, the NHHR and AIP were positively correlated with AHI. However, they were negatively correlated with the lowest SpO_2_. Secondly, the binary logistic regression model indicated that NHHR and AIP were independent factors for OSA. Finally, based on ROC analysis, the NHHR combined AIP exhibited respectable OSA discriminating power. Collectively, these findings highlight a significant relationship between OSA and both the NHHR and the AIP.

Prior research has mostly concentrated on the functions of TC and HDL-C in relation to OSA. A prospective cohort research study showed a significant relationship between HDL-C levels and the severity of sleep-disordered respiration (20). Lei L et al. also found that OSA patients had greater TC and lower HDL-C values (21). A meta-regression study also showed that blood TC, LDL-C, and TG levels rose while HDL-C levels fell in OSA patients (22). Similarly, in our study, in the OSA group, the HDL-C were significantly lower than those in the non-OSA group. These findings collectively suggest complex interrelationships between dyslipidemia and OSA pathogenesis. Nevertheless, extant literature presents notable inconsistencies that warrant further clarification. For example, a cross-sectional analysis demonstrated that no significant association between OSA severity and HDL-C concentrations (23). Similarly, a Mendelian randomization study found no compelling evidence supporting a causal relationship between HDL-C levels and OSA risk (24). We propose that the discrepancies observed across studies may be attributed to several methodological and clinical factors, including heterogeneity in disease severity at enrollment, differences in medication regimens, limitations in sample size, and variations in ethnic composition of study cohorts. The NHHR represents a novel composite biomarker for assessing atherogenic lipid profiles. Previous investigations have demonstrated that NHHR shows superior predictive capability for cardiovascular and metabolic disorders compared to conventional single lipid parameters and established metabolic indices (24, 25). Prior studies have highlighted the association and predictive value of NHHR with several conditions, such as depression (26), kidney stones (27), and suicidal ideation (28). Thus, this study aimed to introduce NHHR as a novel atherosclerosis indicator, potentially enhancing the predictive ability of HDL-C and non-HDL-C for OSA risk. In this study, the NHHR was significantly higher in OSA patients than in non-OSA patients. Furthermore, the NHHR was positively correlated with AHI and was negatively correlated with the lowest SpO_2_. A binary logistic regression model indicated that the NHHR was identified as an independent factor for OSA, which is consistent with previous research (10, 13). This finding suggests that NHHR plays an important role in OSA pathogenesis.

There are several explanations for the correlation between OSA and NHHR. First, chronic intermittent hypoxia (CIH) is the primary contributor to OSA and disturbances in lipid metabolism (29). Hypoxia-inducible factor-1 can be upregulated by CIH, which increases TC biosynthesis (30). The risk of OSA is greatly increased by obesity, and those who are fat typically exhibit more severe abnormalities in their lipid metabolism (1). Excessive adipose tissue can also result in respiratory collapse and obstruction by causing upper airway stenosis, decreased vital capacity, ventilation-perfusion mismatch, and restricted lung and chest wall motion (31, 32). Third, CIH induces an oxidative stress response, resulting in the production of oxidized and dysfunctional lipids (33). Multiple studies have established that non-HDL-C, which encompasses all apolipoprotein B-containing atherogenic lipoproteins, demonstrates stronger associations with cardiovascular risk than LDL-C alone (34, 35). In contrast, HDL-C exerts atheroprotective effects through reverse cholesterol transport (36). Consequently, an elevated NHHR reflects a disproportionate increase in atherogenic lipoproteins relative to protective lipoproteins. This lipid profile imbalance may contribute to OSA pathogenesis by promoting systemic inflammation and endothelial dysfunction. Finally, OSA can potentially induce an increase in sympathetic nervous system activity and influence the production of HDL (37, 38).

Only a small number of research studies have examined the TG/HDL-C ratio or its logarithm in relation to OSA. The association between OSA and elevated AIP has been demonstrated in prior research, but only in subjects of normal weight (39). The TG/HDL-C ratio was correlated with the severity of the disease and was associated with OSA (40). However, a previous investigation demonstrated that TG/HDL-C ratio showed no significant correlation with any sleep quality parameters and was not associated with daytime sleepiness (41). The AIP, calculated as log (TG/HDL-C), represents a unique composite lipid indicator that may more accurately reflect the balance between atherogenic and anti-atherogenic lipid particles (42). Previous studies have shown a significant relationship between AHI and AIP (43, 44). Nonetheless, its clinical significance is constrained (11). In our study, the AIP was significantly higher in OSA patients than in non-OSA patients. Furthermore, the AIP was positively correlated with AHI and was negatively correlated with the lowest SpO_2_. A binary logistic regression model indicated that the AIP was identified as an independent factor for OSA, which is consistent with previous research (43, 44). It was hypothesized that three primary mechanisms may underlie the association between OSA severity and AIP. Firstly, the AIP serves as a reliable indicator of atherogenic dyslipidemia, a condition characterized by elevated TG, reduced HDL-C, and increased levels of small dense low-density lipoprotein (sdLDL)-an LDL subfraction with enhanced pro-inflammatory and pro-atherogenic properties (45). Pathophysiologically, triglyceride-rich lipoproteins (e.g., very-low-density lipoprotein and chylomicrons) possess sufficiently small particle sizes to infiltrate the arterial intima (46, 47), initiating a cascade of sustained low-grade inflammation and foam cell accumulation (48). Furthermore, sdLDL promotes excessive generation of reactive oxygen and nitrogen species, thereby aggravating endothelial dysfunction and vascular injury (49). Secondly, CIH raises systemic TG concentrations by upregulating the expression of TG-synthetic enzymes in the liver (50). Endocrine homeostasis is upset by OSA-related sympathetic hyperactivity, which may aggravate the rise in AIP by causing metabolic disturbances driven by catecholamines (51). Thirdly, it has been demonstrated that pharmacologically blocking alpha-1 adrenergic receptors reduces TG accumulation while increasing HDL-C levels (52).

In our study, data from 454 participants were analyzed; however, it is evident that the study population contains a disproportionately high number of male subjects. We attribute this marked sex imbalance simply reflects the well-known higher prevalence of OSA in men (19). Furthermore, we discovered in our study that BMI and male were independent factors that were linked to OSA, consistent with previous finding (19). OSA is more common in males; however, females experience a notable rise in prevalence post-menopause, likely attributed to reduced estrogen levels (53). Consistent with our findings, prior research has shown that obesity is an independent risk factor for OSA (54–56). Risk factors for OSA include older age (8), However, our analysis revealed no significant association between age and OSA. We propose that discrepancies across studies may be attributed to variations in ethnic composition of study populations, differences in sample sizes, and heterogeneity in disease severity distributions.

To evaluate the overall discriminatory value of the NHHR and AIP in differentiating OSA, we employed ROC curves. Our study demonstrated that the NHHR and AIP may effectively differentiate patients with OSA from those without OSA. The NHHR demonstrates better performance than the AIP in identifying patients with OSA. Importantly, the combination of these two lipid parameters shows a synergistic effect, achieving an AUC of 0.763 and indicating a meaningful improvement in OSA detection capability. The combination of these two indications may be more useful in predicting OSA, as this value exceeded that of the separate markers. However, sensitivity for NHHR (46.5%) was low. The combination of NHHR and AIP shows a markedly high specificity of 89.71%, whereas the sensitivity is comparatively low at 47.48%. The combination of NHHR and AIP demonstrated high specificity but modest sensitivity for OSA. Therefore, it is not suitable as a screening tool, where the primary goal is to rule out disease and high sensitivity is paramount. Instead, its high specificity suggests its utility as a diagnostic adjunct. In clinical practice, a positive result from the NHHR+AIP model could be used to strengthen the clinical suspicion of OSA in symptomatic patients, potentially prioritizing them for more definitive diagnostic testing (e.g., polysomnography) in resource-constrained settings.

The following are some of the research’s shortcomings (1): There might be inherent biases because this was a cross-sectional study that exclusively included Chinese patients. Therefore, it must be confirmed in non-Chinese groups, and larger-scale longitudinal cohort studies might be needed for future research (2); compared to female patients, there were more male patients in this study, which could potentially create biases (3); fasting samples were used to acquire the cholesterol values, which may not be the same as non-fasting readings (4); inadequate analysis of several factors that could affect the outcomes, like medication use (5); these findings should be extended cautiously to the general population because only those who were highly suspected of having OSA were included in this study (6); cross-sectional design precludes causal inferences. A longitudinal analysis would clarify whether NHHR/AIP drives OSA progression or vice versa (7); participants were “highly suspected” of OSA, limiting generalizability. Future population-based studies employing community recruitment strategies are needed to enhance the external validity and clinical applicability of these results (8); Statins were excluded, but other lipid-modifying drugs (e.g., fibrates) or antihypertensives were not accounted for; and (9) lack of data on lifestyle factors that influence lipid profiles.

## Conclusion

In conclusion, our research shows that the NHHR and AIP were independently risk factors for OSA and can be used as potential tools for OSA. Furthermore, there may be more predictive value when NHHR and AIP are combined. Nevertheless, further investigations are warranted to validate these observations and to fully elucidate the underlying pathophysiology of OSA.

## Data Availability

The raw data supporting the conclusions of this article will be made available by the authors, without undue reservation.

## Supplementary material

Supplementary Figure 1 Subgroup analyses of AIP, NHHR and OSA.
