# Supplementary figures and images for: Association of non-high-density lipoprotein cholesterol to high-density lipoprotein cholesterol ratio and atherogenic index of plasma with obstructive sleep apnea
Source: Front Psychiatry. 2026 Jan 12;16:1597820. doi: 10.3389/fpsyt.2025.1597820 (PMC12833340; doi:10.3389/fpsyt.2025.1597820)

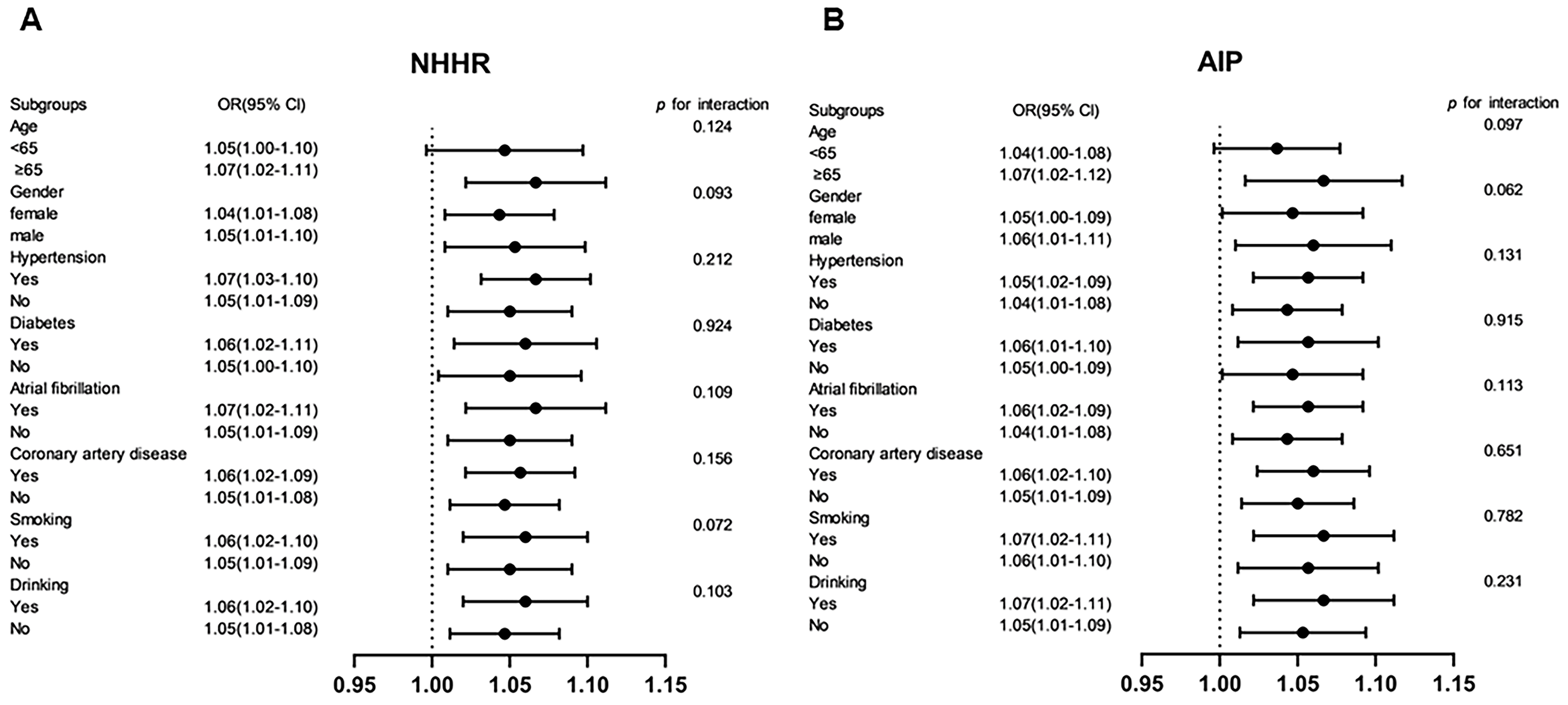

Supplement: Supplementary Figure 1 — Subgroup analyses of AIP, NHHR and OSA. [file Image1.tif]
